# Diseases of the Tonsils

**Published:** 1894-03-03

**Authors:** 


### DISEASES OF THE TONSILS.

*Follicular Tonsillitis.*—E. Fletcher Ingals[1] orders 10 grains of PHEANCETIN every hour for three doses in strong adults. By combining caffeine with it, its depressing effect on the heart may be guarded against when desirable. If it is strongly suspected that the case is of rheumatic origin, the anti-rheumatic remedies are of prime importance.

As to local remedies, he obtains most relief by the application of SILVER NITRATE, 60 grains to the ounce, or of a solution of carbolic acid and tannic water of each 30 grains, and morphine 4 grains, in water and glycerine, half an ounce of each. This causes smarting, but will give the patient great relief for from twelve to eighteen hours, and seems to go far towards checking the inflammation.

*The Tonsils in Scarlet Fever.*—As the result of an elaborate investigation carefully conducted by Dr. Walter Dowson[2] on the role of the tonsil in scarlatinal infection, he is able to bring very strong evidence tending to prove that we should regard the tonsillar lesion characteristic of scarlet fever as the cause rather than the consequence, and not merely symptomatic of a specific septicæmia, or some other circumstance, of the existence even of which no satisfactory evidence is to hand; that, in fine, as in diphtheria, the throat condition and cervical bubo of scarlet fever are the analogues of the chancre and inguinal bubo of syphilis, or of the intestinal ulcers and enlarged mesenteric glands of enteric fever.

*Tonsillotomy.*—The relative infrequency of hæmorrhage following tonsillotomy has been repeatedly discussed of late, and its occasional occurrence used as an argument in favour of substituting for tonsillotomy some methods which are manifestly inconvenient, but which, it is argued, are safer. Mr. P. R. W. de Santi[3] deals fairly exhaustively with the literature of hæmorrhage following tonsillotomy. He quotes Sajous: "When Mackenzie, Browne, Schrötter, Gougenheim, Krause, Massei, and Capart together report 20,000 tonsillotomies with but seven cases of hæmorrhage, the thirty-one cases of bleeding collected by Dr. J. Wright (the result of a search of the records of the previous twenty-five years in the library of his surgeon-general, Washington), would represent at the same ratio about 85,000 tonsillotomies—certainly a strong argument in favour of an operation productive of so much relief." De Santi arrives at the following conclusions.

*What are the Causes of Hæmorrhage after Tonsillotomy?*—(1) Abnormality in the distribution of the bloodvessels of the tonsil; (2) hæmophilia; (3) over use of the voice; (4) the too early eating of solids; (5) the recorded cases of severe hæmorrhage have occurred very frequently after the use of the bistoury.

Nearly all the cases of severe hæmorrhage from tonsillotomy have occurred in adults.

*How Should we Treat the Hæmorrhage?*—Most cases tend to cease spontaneously. The patient should be kept quiet and have small pieces of ice to suck. Should the bleeding persist, a mixture of one part gallic acid to three parts tannic acid dissolved in water may be sipped, or it may be applied to the bleeding tonsil. If these measures fail, then seek for the bleeding point, and, if possible, it should be seized and twisted with torsion forceps, or it may be necessary to touch the bleeding point or surface with a cautery. Failing these and similar expedients, there remains, as a last resource, ligation of one of the carotid arteries, preferably the external carotid.

[1] Int. Clin., Vol. IV., 1893. [2] Med. Chron., Jan., 1894. [3] Lanc., Jan. 13, 1894.

*(To be continued.)*

